# The Diagnostic Utility of Fever in Patients With Suspected Sepsis Secondary to Community-Acquired Bacteremia

**DOI:** 10.7759/cureus.72561

**Published:** 2024-10-28

**Authors:** Phoebe Langius, Donny Perez, Sandra Lopez, Eric Boccio

**Affiliations:** 1 Emergency Medicine, Florida International University, Herbert Wertheim College of Medicine, Miami, USA; 2 Emergency Medicine, Memorial Healthcare System, Hollywood, USA; 3 Emergency Medicine, Memorial Healthcare System, Pembroke Pines, USA

**Keywords:** blood cultures, community-acquired bacteremia, fever, patient-centered outcomes research, sepsis

## Abstract

Background

Community-acquired bacteremia is a potential source of infection and cause of fever in patients presenting to the emergency department (ED) with suspected sepsis. Ambiguous or false-positive blood culture results may lead to unnecessary testing and overtreatment with substantial implications on antimicrobial stewardship and associated healthcare costs. There is little clinical information available to determine with a high degree of certainty whether a patient may be febrile secondary to bacteremia. The primary aim of this study was to assess the diagnostic utility of fever in patients with suspected sepsis secondary to community-acquired bacteremia.

Methodology

A retrospective review of a consecutive sample of electronic health records of patients presenting to the ED of an academic tertiary care hospital with an annual census of approximately 100,000 visits was performed. Structured demographic and clinical data were summarized. Receiver operating characteristics of fever, defined as an initial recorded temperature ≥38°C, in predicting bacteremia, defined as at least one positive blood culture result, were calculated. The associations between fever and bacteremia and admission and inpatient all-cause mortality rates were analyzed using multivariable logistic regression.

Results

A total of 100,270 pediatric and adult ED visits were screened for eligibility. Of the 10,220 (10.2%) patients who had at least one blood culture result and temperature recorded, 1,175 (11.5%) were febrile and 487 (4.8%) had bacteremia. Febrile patients were more likely to have blood cultures drawn than afebrile patients (34% vs. 10%, p < 0.001), and the median initial temperature was higher in patients with a positive blood culture result (37.1°C vs. 36.9°C, p < 0.001). Fever was not sensitive for bacteremia across pediatric, adult, and geriatric cohorts (25.0%, 21.6%, and 23.4%, respectively), but was more specific in adult and geriatric cohorts (90.2% and 92.1%, respectively) than the pediatric cohort (68.6%). A positive blood culture result was associated with an increased likelihood of admission (adjusted odds ratio (AOR) = 2.85, 95% confidence interval (CI) = 2.14-3.81, p < 0.001) and inpatient all-cause mortality (AOR = 3.67, 95% CI = 2.68-5.04, p < 0.001) while fever increased the likelihood of admission only (AOR = 1.58, 95% CI = 1.36-1.83, p < 0.001).

Conclusions

A strict definition of a fever of ≥38°C is specific but not sensitive for bacteremia. Febrile patients are 1.58 times as likely to be admitted when compared to afebrile patients while adjusting for age, sex, and the presence of a positive blood culture result. Bacteremia is associated with increased admission and mortality rates in patients with suspected sepsis presenting to the ED.

## Introduction

Body temperature naturally fluctuates throughout the day and varies depending on age, sex, season, and metabolic, hormonal, and physiological states, but there exists very little research describing how body temperature fluctuates during illness states [1]. The cutoffs for fever in the adult and pediatric populations are clinically defined as temperatures above 38°C (100.4°F) when taken rectally, although other studies have argued for a wider range of the accepted upper limit of normal body temperature [2-4]. The differential diagnosis for a patient presenting to the emergency department (ED) with new-onset fever may initially be broad and is likely to include an underlying infectious process. Based on risk factors, clinical suspicion, and pretest probability, an array of diagnostic tests may be performed depending on the suspected cause.

Bacteremia, or the presence of bacteria in the bloodstream, is assessed through culturing of blood specimens obtained from at least two different sites for the presence of aerobic and anaerobic bacteria. In 2015, the Severe Sepsis and Early Septic Shock Management Bundle (SEP-1) delineated the time-sensitive ordering of blood cultures on patients with severe sepsis and septic shock which led to an increase in blood culture ordering frequency overall and especially in patients discharged from the ED. Results demonstrated a decrease in the rate of positivity for pathogens and an unchanged rate of growth of contaminants suggesting that SEP-1 may have been responsible for an increase ordering of clinically unnecessary blood cultures and perhaps overprescribing of antibiotics [5]. Clinically, the association between fever and bacteremia is not entirely predictable and may vary due to differences in immune responses at the individual patient level [6]. This research sought to assess the diagnostic utility of a strict threshold for fever in patients with suspected sepsis secondary to community-acquired bacteremia and to assess the associations between fever and admission and inpatient all-cause mortality rates and bacteremia and admission and inpatient all-cause mortality rates.

## Materials and methods

Study design and setting

This study was designed as a retrospective review of a consecutive sample of electronic health records of patients presenting to the ED of an academic tertiary care hospital in the northeast United States with a combined annual census of approximately 100,000 visits. This study was approved by the affiliate Institutional Review Board (approval number: 834262-1).

Selection of participants

All patients that presented to the ED over the 12-month period between January 1, 2014, through December 31, 2014, were screened for eligibility. Subjects were classified based on sex, as febrile or afebrile, whether blood cultures were obtained, and whether at least one blood culture result was positive. Subjects were stratified into pediatric, adult, and geriatric cohorts based on age (<18 years, ≥18 years and <65 years, and ≥65 years, respectively).

Measurements

Body temperature was defined as the initial recorded body temperature independent of the method and source. Fever was defined by the strict threshold of initial recorded body temperature greater than or equal to 38°C. Bacteremia was defined as at least one positive blood culture result demonstrating at least a single species exclusive of coagulase-negative staphylococci as these species are commonly considered contaminants.

Outcomes

Receiver operating characteristics including sensitivity, specificity, positive predictive value, and negative predictive value of fever in predicting bacteremia were calculated. Secondary outcomes included admission and inpatient all-cause mortality rates.

Analysis

Structured demographic and clinical information for all subjects were summarized. The associations between fever and admission and inpatient all-cause mortality rates and bacteremia and admission and inpatient all-cause mortality rates while adjusting for demographic covariables were analyzed using multivariable logistic regression. All analyses were conducted using SPSS version 23.0 for Windows (IBM Corp., Armonk, NY, USA). The level of significance was set a priori at 0.05.

## Results

Characteristics of study subjects

A total of 100,270 pediatric and adult ED visits were screened for eligibility. Of the 10,220 (10.2%) patients who had a temperature recorded and at least one blood culture result, 1,175 (11.5%) were febrile and 487 (4.8%) had bacteremia. The median age was 58 years (interquartile range (IQR) = 38-74 years), and 889 (8.7%) were younger than 18 years of age. Females represented 48.2% of all subjects. Febrile patients were more likely to have blood cultures drawn than afebrile patients (34% vs. 10%, p < 0.001), and the median initial temperature was higher in patients with a positive blood culture result (37.1°C vs. 36.9°C, p < 0.001). A summary of subject demographic and clinical characteristics for the overall sample and stratified by age is provided in Table 1.

**Table 1:** Summary of subject demographic and clinical characteristics for the overall sample and stratified by the age group. Higher rates of fever were seen in the pediatric group with similar rates across adult and geriatric groups. Positive blood culture rate appears to increase with increasing age with the lowest rate (2.7%) seen in the pediatric group and the highest rate (6.4%) seen in the geriatric group. IQR: interquartile range

	<18 (n = 889)	18–64 (n = 5,119)	≥65 (n = 4,212)	Total (n = 10,220)
Median age, years (IQR)	4 (0–10)	47 (33–56)	78 (71–85)	58 (38–74)
Female, %	44.8	47.3	50.0	48.2
Fever, %	31.3	10.2	8.9	11.5
Positive blood culture, %	2.7	3.8	6.4	4.8

Main results

**Figure 1:**
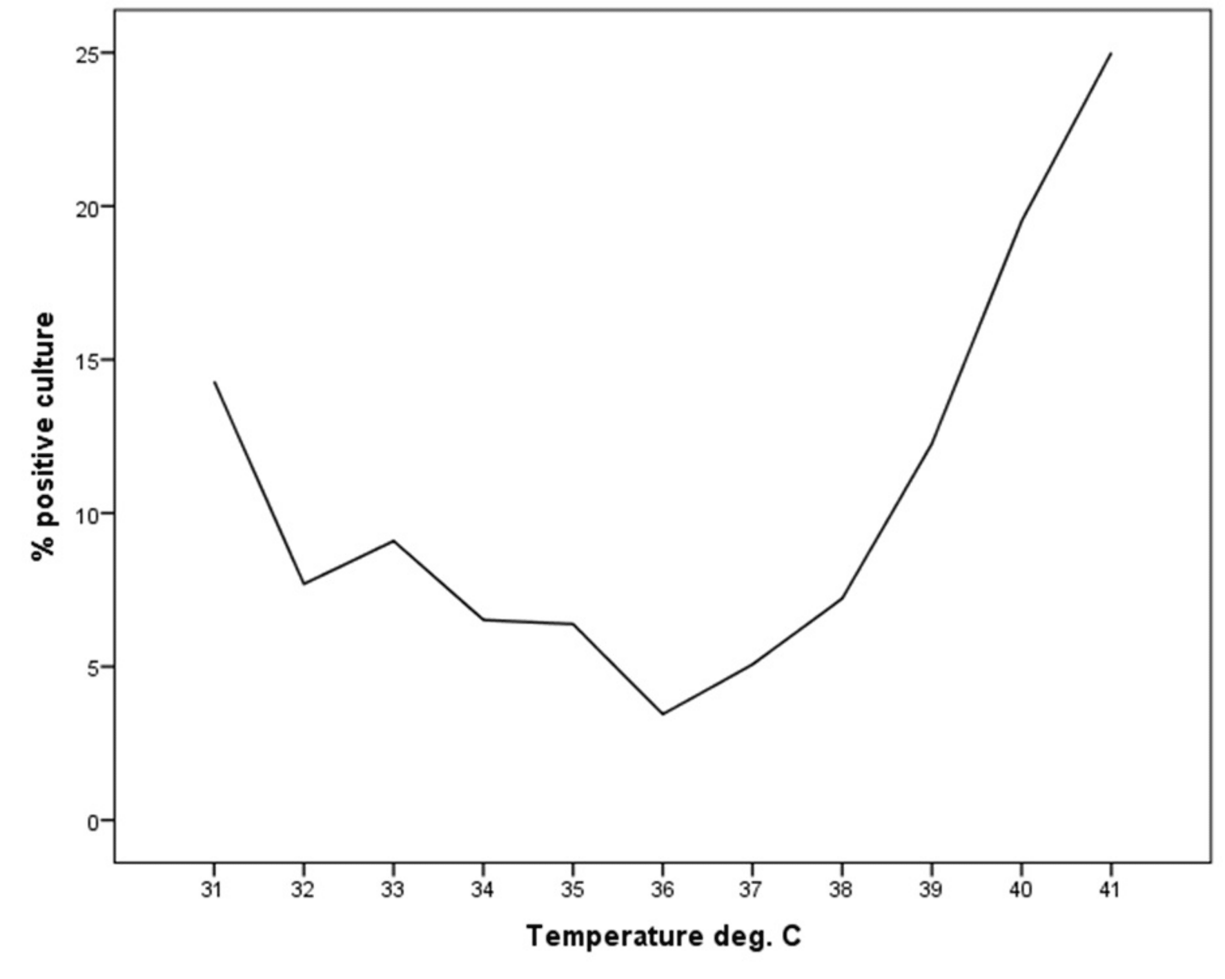
Positive blood culture rate (%) as a function of initial body temperature (°C). The distribution of positive blood culture rate as a function of initial body temperature appears to be quadratic with increasing rates as body temperature decreases and increases about the median. Extremes of body temperature appear to be associated with the highest positive blood culture rates, suggesting that bacteremia may be present in both hypothermic and hyperthermic patients.

Crude percent positive blood culture rate as a function of initial body temperature is illustrated in Figure 1. Fever was not sensitive for bacteremia across pediatric, adult, and geriatric groups (25.0%, 21.6%, and 23.4%, respectively), but was more specific in adult and geriatric groups (90.2% and 92.1%, respectively) than the pediatric group (68.6%) (Table 2).

**Table 2:** Receiver operating characteristics of fever in predicting bacteremia for the overall sample and stratified by age group. Fever is not sensitive for bacteremia across all age groups. Fever appears to be specific for bacteremia in adult and geriatric groups with high negative predictive values across all ages.

Age, years	Sensitivity, %	Specificity, %	Positive predictive value	Negative predictive value
<18	25.0	68.6	2.2	97.1
18–64	21.6	90.2	8.0	96.7
65+	23.4	92.1	16.8	94.6
Total	22.8	89.1	9.4	95.8

Admission and mortality rates were higher for febrile patients than for afebrile patients (31% vs. 22%, p < 0.001, and 1.2% vs. 0.5%, p < 0.001, respectively). Admission and mortality rates were higher in patients who had blood cultures drawn than those who did not (71% vs. 17%, p < 0.001, and 3.1% vs. 0.2%, p < 0.001, respectively). Of patients who had blood cultures drawn, admission and mortality rates were higher in patients with positive blood cultures than with negative blood cultures (89% vs. 70%, p < 0.001, and 10.9% vs. 2.7%, p < 0.001, respectively).

As patient age increased per year, admission and mortality rates also increased (adjusted odds ratio (AOR) = 1.02, 95% confidence interval (CI) = 1.018-1.021, p < 0.001, and AOR = 1.037, 95% CI = 1.031-1.044, p < 0.001, respectively). Stated differently, as age increased by one year, admission and mortality rates increased by approximately 2% and 4%, respectively. Females were less likely to be admitted than males (AOR = 0.85, 95% CI = 0.78-0.93, p < 0.001). Febrile patients were 1.58 times more likely to be admitted than afebrile patients (95% CI = 1.36-1.83, p < 0.001), but the association between fever and mortality did not reach statistical significance. Positive blood culture was the strongest predictor of admission and inpatient mortality (AOR = 2.85, 95% CI = 2.14-3.81, p < 0.001, and AOR = 3.67, 95% CI = 2.68-5.04, p < 0.001, respectively) (Table 3).

**Table 3:** Results of multivariable regression analysis for the outcomes of admission and inpatient all-cause mortality while adjusting for demographic and clinical covariates. Covariables found to have statistically significant associations (p-value <0.05) with admission and inpatient all-cause mortality and their respective adjusted odds ratios and 95% confidence intervals are provided. Fever, positive blood culture, and age appear to increase the likelihood of admission while the female sex is protective. Positive blood culture and age are both associated with an increased likelihood of inpatient all-cause mortality. Positive blood culture appears to represent the highest risk of both inpatient admission and all-cause mortality. AOR: adjusted odds ratio; CI: confidence interval

Admission	AOR	95% CI	P-value
Fever	1.58	1.36–1.83	<0.001
Positive blood culture	2.85	2.14–3.81	<0.001
Age (per year)	1.020	1.018–1.021	<0.001
Female	0.85	0.78–0.93	<0.001
Inpatient mortality			
Positive blood culture	3.67	2.68–5.04	<0.001
Age (per year)	1.037	1.031–1.044	<0.001

## Discussion

The Centers for Medicare and Medicaid Services introduced SEP-1 as a national quality measure in October 2015 to standardize care and improve clinical outcomes of patients presenting along the spectrum of sepsis severity. The SEP-1 metric bundle includes the collection of culture specimens before the administration of antibiotics to confirm the suspected source of infection and aid in the identification of microbial species and antimicrobial sensitivities [7]. This information supports targeted antimicrobial therapy, thus preventing the promotion of resistant organisms and minimizing patient side effects while also providing prognostic value [8]. Two sets of blood cultures are taken from two different sites and a sterile technique must be maintained to prevent contamination from the patient and provider’s skin flora [9].

The differential diagnosis of febrile illness typically includes bacteremia, and the pretest probability may vary based on individual patient-level risk factors and histories [10]. Our results demonstrate that approximately one-third of patients with documented fever had a blood culture ordered, whereas 10% of afebrile patients had a blood culture ordered. This comparison of blood culture ordering rates between febrile and afebrile patients demonstrates the clinical significance of fever in the provider’s decision to order a blood culture. Previous studies have shown that physicians tend to overestimate the likelihood of bacteremia in patients with febrile illness, and, thus, a higher percentage of blood culture ordering in febrile patients was expected [11]. We decided to assess ordering practices by emergency medicine providers before the implementation of SEP-1 to control for possible external influences of metric compliance. Despite the high rates of blood culture ordering, few of these blood cultures yield true-positive results [9,12]. Our results demonstrated a low rate of positive blood cultures for non-coagulase-negative staphylococci (4.2%). Low diagnostic yield becomes further problematic when considering the pain caused to the patient when performing a second needle stick; the time and financial costs associated with collecting, storing, and growing the specimens; and the implications unnecessary additional testing and overtreating have on potentiating adverse side effects and antimicrobial resistance patterns when providers respond to ambiguous or false positive results. On the other hand, the all-cause mortality rate was 3.67 times higher in admitted patients with a positive blood culture compared to those with a negative blood culture, when adjusting for demographic covariables. The high mortality rate (8%) associated with community-acquired bacteremia underscores the importance of identifying patients at risk and justifies quality measures surrounding blood culture ordering. These factors may translate into a lower risk acceptance threshold which may be a secondary explanation for the high rates of blood culture ordering observed in the ED and emphasizes the need for a better diagnostic approach to bacteremia.

The median initial temperatures between patients with positive and negative blood culture results were almost identical (37.1°C vs. 36.9°C, respectively). Despite reaching statistical significance, the difference in initial temperature is not obviously clinically significant and raises the question as to whether fever should be considered a strict clinical criterion in the decision to order a blood culture across all patient subtypes. Patients at the extremes of age and with comorbidities such as cirrhosis or differences in immunocompetency may not exhibit a rise in body temperature when exposed to bacteremia due to a blunted immune response [10]. As a result, the traditionally accepted thresholds for diagnosing fever may not apply to all patients, and careful consideration is needed for the hypothermic patient. Patients with a documented fever had higher admission and inpatient mortality rates than patients without (31% vs. 22%, p < 0.001, and 1.2% vs. 0.5%, p < 0.001, respectively). Patients who had a blood culture ordered had higher admission and inpatient mortality rates than those who did not (71% vs. 17%, p < 0.001, and 3.1% vs. 0.2%, p < 0.001, respectively). These results may be explained by the fact that febrile patients appear to be sicker and thus require a broader workup and more aggressive treatment which often results in admission and perhaps ultimately inpatient mortality. The association between blood culture ordering and increased hospitalizations has been described in other pediatric populations [13]. The fact that 29% of patients who had blood cultures ordered were discharged from the ED before results were available raises the question as to why they were ordered in the first place and whether providers are overordering cultures in general. Culture results do not appear to be disposition-dependent for approximately one-third of ED patients, and cultures are certainly not indicated as part of the workup of fever of suspected viral etiology when the pretest probability of bacterial infection is sufficiently low [14]. Emerging diagnostic biomarkers and devices to differentiate bacterial and viral infections to increase diagnostic certainty may prove beneficial in reducing the judicious ordering of cultures and overprescribing of antibiotics [15].

As this study involves a retrospective review of electronic health records, the investigators could not control exposure or outcome assessment. It must be assumed that healthcare providers involved in the direct care of subjects included in the analysis were able to accurately document demographic and clinical data as information bias arising from measurement error may occur. The site of body temperature measurement and use of antipyretics before ED arrival was unknown, and no adjustments were made for comorbidities, immunocompetency, or confirmed source of infection beyond a positive blood culture result. The results of this study are unable to provide specific recommendations when there is a suspicion of endocarditis. Fever was defined as an initial body temperature greater than 38°C, and fevers documented after the initial triage vital signs and the presence of hypothermia were not accounted for. Nursing and phlebotomist sterile technique was not standardized, and contaminants may have been included as false-positive blood culture results. The authors attempted to address this issue by considering all coagulase-negative staphylococci species as contaminants. All data was gathered from patient visits to a single center which may limit the generalizability of results. Bias may stem from individual provider practices and opinions surrounding the management of febrile patients and the ordering of blood cultures.

## Conclusions

The strict definition of fever as a body temperature greater than 38°C at initial presentation to the ED does not appear to be a sensitive measure of community-acquired bacteremia across age groups; however, it is fairly specific in adult and geriatric populations with associated negative predictive values close to 95%. With the challenges of interpreting ambiguous blood culture results, increased healthcare costs associated with unnecessary testing and overtreatment, and detrimental effects on antibiotic stewardship and microbial resistance, the authors encourage conservative ordering of blood cultures in cases where adult and geriatric patients are afebrile and there is a low pretest probability of bacteremia. However, given the poor sensitivity and significantly increased risk of mortality in patients with community-acquired bacteremia, blood cultures should be obtained in all febrile patients and afebrile pediatric patients when the pretest probability supports testing. Further investigation to develop more effective diagnostic and clinical decision-support tools to predict community-acquired bacteremia is warranted.
